# Impact of economic crises on mental health care: a systematic review

**DOI:** 10.1017/S2045796018000641

**Published:** 2018-11-13

**Authors:** M. Silva, D. M. Resurrección, A. Antunes, D. Frasquilho, G. Cardoso

**Affiliations:** 1 Chronic Diseases Research Center (CEDOC), NOVA Medical School, Faculdade de Ciências Médicas, NOVA University of Lisbon, Lisbon, Portugal; 2 Universidad Loyola Andalucía, Seville, Spain

**Keywords:** Economic crisis, mental health care, systematic review, use of service

## Abstract

**Aims:**

Unmet needs for mental health treatment are large and widespread, and periods of economic crisis may increase the need for care and the treatment gap, with serious consequences for individuals and society. The aim of this systematic review was to summarise the empirical evidence on the association between periods of economic crisis and the use of mental health care.

**Methods:**

Following the PRISMA statement, MEDLINE, Embase, Scopus, Open Grey and Cochrane Database were searched for relevant publications, published between 1990 and 2018, from inception to June 2018. Search terms included (1) economic crisis, (2) use of mental health services and (3) mental health problems. Study selection, data extraction and the assessment of study quality were performed in duplicate.

**Results:**

Seventeen studies from different countries met the inclusion criteria. The results from the included articles suggest that periods of economic crisis might be linked to an increase of general help sought for mental health problems, with conflicting results regarding the changes in the use of specialised psychiatric care. The evidence on the use of mental health care specifically due to suicide behaviour is mixed. The results also suggest that economic crises might be associated with a higher use of prescription drugs and an increase in hospital admissions for mental disorders.

**Conclusions:**

Research on the impact of economic crises on the use of mental health care is scarce, and methodologies of the included papers are prone to substantial bias. More empirical and long-term studies on this topic are needed, in order to adapt mental health care systems to the specific needs of the population in times of economic crisis.

## Introduction

The financial crisis that hit the global economy in 2008 led to the deepest recession since the 1930s (European Commission, 2009), possibly longer, wider and deeper than the Great Depression (Bambra *et al*., 2016). The crisis had a varied impact across countries, resulting in a decline in gross domestic product (GDP), a rise in unemployment rates and severe fiscal pressure (Thomson *et al*., 2014). Many countries adopted austerity policies, with substantial reductions in public spending affecting health and social care budgets, and many citizens faced growing insecurity and social exclusion (Thomson *et al*., 2014).

Research on the social determinants of mental health has shown that health is shaped by social and economic conditions, as well as by health and welfare systems (World Health Organization and Calouste Gulbenkian Foundation, 2014). Economic crises may affect mental health either by increasing risk factors, such as unemployment, indebtedness and loss of socioeconomic status, or by weakening protective factors, such as job security and welfare protection programmes (Caldas de Almeida *et al*., 2017). Indeed, recent reviews assessing the health consequences of economic crises have revealed a significant relationship between these periods and psychopathology including suicide, onset or exacerbation of mood and anxiety disorders, heavy drinking, and psychological distress (Frasquilho *et al*., 2016; Martin-Carrasco *et al*., 2016). These results would make expectable an increased search for mental health treatment. However, barriers to access to mental health care may be exacerbated during economic crises, due to changes in the availability (e.g., cuts in human resources) and affordability (e.g., out-of-pocket payments) of services (Wahlbeck and McDaid, 2012; Maresso *et al*., 2015; Antunes *et al*., 2017). Literature on how the use of mental health care varies in times of economic crisis is scarce, and recent reviews found mixed evidence (Zivin *et al*., 2011; Cheung and Marriott, 2015; Martin-Carrasco *et al*., 2016). Zivin *et al*. (2011) concluded that economic downturns might be associated with increased first admissions to mental health services. Cheung and Marriott (2015) found a decline in the use of mental health services in the USA, likely due to a lack of access to insurance, and an increase in the use of prescription medication. Martin-Carrasco *et al*. (2016) concluded that the treatment gap increases in times of economic crisis, probably due to the lack of accessibility to services, the austerity measures, and the increased stigma towards people with mental illness. However, these reviews had some limitations: (a) only one of them followed the PRISMA guidelines (Martin-Carrasco *et al*., 2016); (b) one of the systematic reviews did not focus specifically on the use of mental health care (Martin-Carrasco *et al*., 2016); and (c) one did not include data from the 2008 Great Recession (Zivin *et al*., 2011).

The aim of this study was to systematically review the available literature on the impact of economic crises on the use of mental health care, information that might contribute to the design of strategies, policies and programmes to promote equitable access to care in times of economic crisis.

## Methods

### Search strategy and selection of articles

The PRISMA guidelines for reporting systematic reviews were followed (Moher *et al*., 2009). The protocol was registered in the International Prospective Register of Systematic Reviews on 28 June 2017 (PROSPERO, registration No.: CRD42017069284). Comprehensive literature searches of MedLine (through Ovid and Pubmed), Scopus, Cochrane Database and Open Grey Repository databases were conducted, from inception to 20 June 2017 and last updated on 25 June 2018. Databases were searched separately by two reviewers (DMR and MS).

Three sets of keywords were combined in the search strategy: (1) economic crisis; (2) use of mental health; (3) mental health problems. Searches were piloted in Ovid and then adapted to run across the other databases (see Supplementary Table S1). The reference lists of the primary studies selected as well as recent reviews in the field were checked. In addition, we contacted expert authors to identify any additional articles.

Study selection was done in duplicate (DMR and MS), and a third reviewer participated where disagreements arose (GC) over the three phases. First, duplicate studies were deleted. Second, a selection of potentially relevant articles was made based on the title and abstract. Third, after reading the full text, a final selection was made. The inter-agreement between reviewers measured with the *κ* statistic was excellent (*κ* = 0.81; 95% CI 0.65–0.97).

The studies selected had to meet specific inclusion criteria (see Table 1). We focused on countries that faced crises since the 1990s as this would allow the inclusion of the available research on the impact of the main economic crises on health, namely the Post-Communist Depression in the early 1990s, the East Asian financial crisis in the late 1990s, and the Great Recession in 2008. We selected only studies with a predominantly adult population, excluding those focusing on children and adolescents, as differences in psychopathology, clinical and social characteristics between children/adolescents and adults would make it difficult to draw conclusions. We excluded studies focusing on residential care, due to the fact that during economic crises the population living in residential care, although vulnerable, were likely to be less exposed to factors affecting an individual's search for mental health treatment (social determinants of mental health and barriers to treatment) compared with those residing in permanent private dwellings, and it would be difficult to make comparisons between the two populations. Only observational studies, including ecological, cross-sectional, case–control and longitudinal studies, were selected. We included all health settings which were accessed with mental health problems as the main complaint.

**Table 1.** Inclusion and exclusion criteria for the studies included in the systematic review

Aspects considered	Inclusion criteria	Exclusion criteria
Population	Adult population with any mental health problem/disorder; countries that faced crises since the 90s	Population not accessing health care for mental health problems; population with a specific medical condition
Outcome	Access/use of mental health care (visits, admissions, lengths of stay, emergencies); use of psychotropic medication; referral to specialised psychiatric care	Impact on services (budget, organisational, financial); focused on cost; impact only on mental health prevalence
Design	Observational studies, including ecological, cross-sectional, case–control and longitudinal studies	Randomised controlled trials, systematic reviews, meta-analysis, editors’ letters, clinical cases, protocols, qualitative studies
Language	All	None
Setting	Primary care; psychiatric/mental health outpatient services; psychiatric/mental health inpatient services	Non-psychiatric care; residential care

### Summary measures

The summary of measures included in the selected studies were relative risk (RR), adjusted relative risk (ARR), adjusted incidence rates (AIR) and incidence risk ratio (IRR).

### Data synthesis

We developed a data extraction sheet, pilot tested it on three randomly selected studies that had been included and refined herein. The main characteristics of these studies were rigorously extracted by MS and verified by a second reviewer (DMR). Any discrepancies were resolved by discussion between the two reviewers. In the event of disagreement, a third reviewer (GC) was consulted.

For each study, information was collected about the author(s), year of publication, study country, setting, sample size, time period of crisis, study design, purpose of the study, outcome variable (indicator), procedure for data collection and main results (see Table 2).

**Table 2.** Details of studies included in this systematic review

Author, year, country	Setting/population	Time period considered	Study design	Purpose of the study	Indicator/outcome considered	Procedure for data collection	Main results
Ásgeirsdóttir *et al*., 2017 Iceland	National population sample, general hospital, *n* = 2548 (41% men, 59% women)	2003–12	Repeated cross-sectional study	To examine potential changes in suicide attempts and self-harm in Iceland during a period of major economic transition	Incidence of attendance rates due to suicidal behaviour	Hospital records	Risk of attendance post-collapse compared with pre-collapse (95% CI): Total sample: RR = 0.95 (0.90–1.01) Women Total sample: 0.97 (0.89–1.04) 18–25 years: 0.95 (0.82–1.1) 26–35 years: 1.16 (0.99–1.37) 36–45 years: 0.87 (0.73–1.03) 46 + years: 0.94 (0.82–1.08) Men Total sample: 0.94 (0.86–1.03) 18–25 years: 1.00 (0.83–1.19) 26–35 years: 0.83 (0.69–1.00) 36–45 years: 0.82 (0.67–1.00) 46 + years: 1.13 (0.94–1.34)
Bidargaddi *et al*., 2015 Australia	Regional population sample, emergency services, *n* = from 978 to 1421 per month (mean 1226.5; s.e. 10.11)	2004–11	Time series	To analyse the effects of changes in rates of unemployment on numbers of Emergency Department Mental Health presentations	Use of emergency department due to mental health problems	Hospital records	Cross-correlation between unemployment and mental health presentations to emergency departments: At current month: CC = 0.22; s.e. = 0.2 A lag of 2 months: CC = 0.36; s.e. = 0.2 Men: 2 months prior unemployment rates: CC = 0.36; s.e. = 0.2 Women: At current month: CC = 0.23; s.e. = 0.2 Previous months: CC = 0.29; s.e. = 0.2 Cross-correlation between men's unemployment and mental health presentations of women: Lag 2 of men's unemployment: CC = 0.24; s.e. = 0.2
Bonnie Lee *et al*., 2017 Taiwan, Republic of China	National population sample, hospitals, *n* = 11 643 417 (53% men and 47% women)	2007–2012	Time series	To evaluate the impacts of the 2008 financial crisis on different socioeconomic subgroups in Taiwan	Monthly adjusted incidence rates of hospitalisation	Hospital records	Effects of economic recession in change (in percent) on incidence rates of hospitalisation due to depressive illnesses (per 1 000 000 persons): Men Low income: AIR = 18.01 (95% CI 14.53–21.48) Middle income: AIR = −3.56 (−1.93 to –5.18) High income: AIR = 2.76 (−7.56 to 13.09) Women Low income: AIR = 14.23 (8.46–19.99) Middle income: AIR = −0.28 (−4.95 to 4.39) High income: AIR = 5.02 (4.05–6.00)
Buffel *et al*., 2015 European countries	National population samples, general and mental health outpatient visits, *n* = 52 216 (45% men, 55% women)	2002, 2005–2006, and 2010	Repeated cross-sectional study	To examine whether the macro-economic context and changes therein are related to mental health care use, via their impact on mental health, or more directly, irrespective of mental health	Use of mental health care	Structured interviews	General practitioner consultations^1^: Women 2002: OR = 0.728*** 2010: OR = 1.118* Men 2002: OR = 0.708* 2010: OR = 1.022 Psychiatrist consultations^1^: Women 2002: OR = 0.850* 2010: OR = 0.879* Men 2002: OR = 0.916 2010: OR = 0.966 *p < 0.05; ***p < 0.001 ^1^Adjusted for the interaction effects with the non-employed
Burgard and Hawkins, 2014 USA	National population sample, all settings, *n* = 73 403	2006–2010	Repeated cross-sectional study	To examine the consequences of the Great Recession for disparities in foregone care in the USA	Foregone use of mental health care	Structured interviews	Predicted percent reporting foregone mental health care by recessionary period: Prerecession: 2.81%; *p* > 0.05 Early recession: 3.36%; *p* = 0.006 Recession and postrecession: 3.39%; *p* = 0.001
Chen and Dagher, 2016 USA	National population sample, primary care and hospital outpatient visits, *n* = 23 317 (29% men, 71% women)	2007–2009	Longitudinal study (panel study)	To examine the changes in health care utilisation for mental health disorders among patients who were diagnosed with depressive and/or anxiety disorders during the Great Recession in the USA	Utilisation of prescription drugs. Use of mental health care	Medical records	Negative binomial results of differences in prescription drug use and physician visits before and during the recession: Women Prescription drug use 2007: IRR = 1.07; *p* = 0.23 2008: IRR = 1.20; *p* ⩽ 0.001 2009: IRR = 1.20; *p* ⩽ 0.001 Physician visits 2007: IRR = 0.94; *p* = 0.68 2008: IRR = 1.28; *p* = 0.05 2009: IRR = 1.03; *p* = 0.83 Men Prescription drug use 2007: IRR = 0.98; *p* = 0.79 2008: IRR = 1.13; *p* = 0.19 2009: IRR = 1.11; *p* = 0.25 Physician visits 2007: IRR = 0.66; *p* = 0.06 2008: IRR = 0.74; *p* = 0.16 2009: IRR = 0.55; *p* ⩽ 0.001
Córdoba-Doña *et al*., 2014 Spain	Regional population sample, emergency services, *n* = 24 380 (47% men, 53% women)	2003–2012	Ecological study	To examine the impact of the economic crisis on suicide attempts, and its relation to unemployment, age and sex	Use of emergency departments due to suicide behaviour	Hospital records	Linear regression fixed-effects models for suicide attempt rates (×10^5^) regressed on unemployment rates^1^: Men *β* = 1.08, *p* = 0.04 (95% CI 0.06–2.09) Women *β* = 0.49, *p* = 0.52 (−1.23 to 2.21) ^1^Adjusted for yearly trends
Dunlap *et al*., 2016 USA	National population sample, outpatient or inpatient mental health treatment, *n* = 21 100 (49% men, 51% women)	2008–2010	Repeated cross-sectional study	To examine the relationship between state and local economic conditions and serious psychological distress, substance use disorders, and mental health service utilisation	Mental health service utilisation	Structured interviews	Predictors of mental health service utilisation: Serious mortgage delinquency rate (quartile) (reference: quartile 1) Quartile 2: ARR = 0.73 (95% CI 0.55–0.98) Quartile 3: ARR = 0.52 (0.38–0.71) Quartile 4: ARR = 0.54 (0.36–0.82) County unemployment rate (quartile) (reference: quartile 1) Quartile 2: ARR = 0.71 (0.60–0.84) Quartile 3: ARR = 0.62 (0.52–0.73) Quartile 4: ARR = 0.58 (0.46–0.74)
Gotsens *et al*., 2015 Spain	National population sample, all settings, *n* = 23 760 in 2006 and *n* = 16 616 in 2012	2006 and 2012	Repeated cross-sectional study	To analyse health inequalities between immigrants born in middle- or low-income countries and natives in Spain, in 2006 and 2012	Utilisation of prescription drugs	Structured interviews	Use of psychotropic drugs: PR 2006 = 0.22 (95% CI 0.11–0.43) PR 2012 = 1.20 (0.73–2.01)
Hawton *et al*., 2016 UK	Regional population sample, general hospitals, *n* = 35 951 (40% men, 60% women)	2008–2010	Ecological study	To investigate the impact of the recent recession on rates of self-harm in England and problems faced by patients who self-harm	Use of emergency departments due to suicide behaviour	Hospital records	Estimates of changes in rates of self-harm in 2008–2010 relative to expected rates based on trends in 2001–2007: Self-harm Oxford Men: 6 (95% CI –2 to 14) Women: −9 (−26 to 8) Manchester Men: 16 (3–30)* Women: −2 (−20 to 16) Derby Men: 17 (1–33)* Women: 35 (18–52)* *p < 0.05
Iglesias García, 2014 Spain	Regional population sample, general hospitals and mental health outpatient visits, *n* = 1 078 406 (48% men, 52% women)	2000–2010	Ecological study	To study the association between socioeconomic status and number of people demanding mental health services	Annual incidence and prevalence demand for mental illness	Hospital records	Administrative incidence of mental disease per 1000 inhabitants: *Z* = −1.863; *p* = 0.03
Korkeila *et al*., 1998 Finland	National population, psychiatric hospitals, *n* = 24 546 and *n* = 24 909 (53% men, 47% women)	1900–1993	Retrospective cohort study	To study the changes in psychiatric inpatient population after (1) the rapid reduction in the number of beds in psychiatric hospitals, (2) the amendment of the mental health legislation and (3) the economic recession	Discharges from psychiatric hospitals	Hospital records	Readmissions to psychiatric hospitals (Poisson regression analysis) First-timers: N° readmissions: 9616 (1991), 9361 (1994); *p* = 0.003 3 or more readmissions: 267 (1991), 402 (1994); *p* < 0.001 All patients: N° readmissions: 15 964 (1991), 15 715 (1994); *p* = 0.005 3 or more readmissions: 1407 (1991), 1796 (1994); *p* < 0.001 Total: 24 546 (1991), 24 909 (1994); NS
Modrek *et al*., 2015 USA	National population sample, outpatient physician visits (including emergency room visits) and inpatient hospitalisations, *n* = 11 625 (80% men, 20% women) and *n* = 10 242 (81% men, 19% women)	2007–2012	Cohort study	To examine the mental health effects of the Great Recession of 2008–2009 on workers who remained continuously employed and insured	Use of mental health care. Utilisation of prescription drugs	Medical records	Mental health inpatient utilisation: *β* = 0.002; *p* = 0.078 Mental health outpatient utilisation: *β* = 0.019; *p* < 0.001 Mental health medication supply (in 2007/2010): Opiates: *β* = 0.777; *p* = 0.057 Antidepressants: *β* = 3.54; *p* < 0.001 Sleep aids: *β* = 0.997; *p* < 0.01 Mental health medication supply (in 2007/2012): Opiates: *β* = 0.072; *p* > 0.05 Antidepressants: *β* = 2.344; *p* < 0.01 Sleep aids: *β* = 0.685; *p* < 0.05
Ostamo and Lönnqvist, 2001, Finland	Regional population sample, general hospitals, *n* = 3100	1989–1997	Time series	To investigate the rates and trends of attempted suicide treated in health care during a period of severe economic recession	Use of health care due to suicide behaviour	Structured interviews and medical records	Trends of attempted suicide: Men: *χ*^2^ = 10.4461; *p* = 0.0012 Women: *χ*^2^ = 2.553; *p* = 0.11
Petrou, 2017, Cyprus	Regional population sample, primary care and hospital outpatient visits	2011–2014	Time series	To elucidate the impact of crisis and introduction of copayment in the utilisation of mental health services in Cyprus primary public health care sector	Use of mental health care	Medical records	Visits to mental health services: Non-significant increase during the early phases of the financial crisis; *p* = 0.978. Introduction of copayment: No impact on mental health services utilisation rate, up to 9 months following its inception; *p* = 0.097
Sicras-Mainar and Navarro-Artieda, 2015 Spain	Regional population sample, primary care and hospital outpatient visits, *n* = 3662 and *n* = 5722	2008–2009 and 2012–2013	Retrospective, observational study	To describe antidepressant use in the treatment of major depressive disorder during a period of economic crisis	Utilisation of prescription drugs. Use of mental health care	Medical records	Total pharmaceutical spending and consumption of antidepressants during the two study periods: Precrisis period (2008–2009)/period of economic crisis (2012–2013) Amount (mg): 1 032 494 352/2 306 366 425 Amount/defined daily dose: 26 728 274/56 446 109 Spending: 1 436 924/1 376 120 Defined daily dose/patient: 7298.8/9864.8 Description of persistence, treatment strategies, referrals to specialist care and use of resources during the two study periods: Precrisis period/period of economic crisis Number of patients *N* (%): 3662 (39.0)/5722 (61.0) Persistence with treatment Average, 6 months: 74.3% (95% CI 72.9–75.7%)/73.5% (95% CI 72.4–74.6%); *p* = 0.371 Average, 12 months: 49.7% (95% CI 48.1–51.3%)/51.8% (95% CI 50.5–53.1%); *p* = 0.002 Referrals to a specialist Rate of referrals: 20.3% (95% CI 19.0–21.6%)/23.8% (95% CI 22.7–24.9%); *p* < 0.001 Use of resources Average number of primary care visits: 8.2 (7.2)/9.9 (7.4); *p* < 0.001 Average number of visits to a specialist: 2.5 (1.8)/2.2 (1.7); *p* = 0.225 Values are expressed as percentage or mean (mean [s.d.])
Wong *et al*., 2014, USA	National population sample, primary care and outpatient visits, *n* = 11 041 855	2004–2012	Longitudinal study	To examine whether local unemployment was associated with utilisation of Veterans Affairs Health Care System primary care, specialty care and mental health services during 2004–2012	Use of mental health care	Medical records	Mental health visits at baseline and study end: Under 65 Fiscal year 2004–quarter 1: mean 2064.32, s.d. 3688.00 Fiscal year 2012–quarter 4: mean 3073.44, s.d. 4886.73; *p* < 0.001 65+ Fiscal year 2004–quarter 1: mean 358.65, s.d. 579.86 Fiscal year 2012–quarter 4: mean 743.89, s.d. 1040.69; *p* < 0.001 Association between local area unemployment rates and outpatient utilisation, by copayment status and age group: For copayment-exempt veterans under age 65, a 1% increase in the local area unemployment rate (LAUR) was associated with a 2.48% (95% CI 2.25–2.70%) increase in average mental health visits For age 65+ copayment-exempt veterans, the LAUR was associated with increases in mental health (2.11%; 95% CI 1.82–2.39%) visits For veterans subject to copayments and under age 65, the LAUR was not significantly associated with mental health visits (0.05%; 95% CI 0.35–0.45%) Among age 65+ veterans subject to copayments, the LAUR was not significantly associated with clinic-level mental health visits (0.28%; 95% CI 0.37–0.93%)

AIR, adjusted incidence rates; CC, cross-correlation coefficient; PR, prevalence ratio; s.e., standard error.

### Risk of bias in individual studies

Quality assessment was performed independently in duplicate (DMR and MS), and a third reviewer participated in cases of disagreement (GC). The quality of the studies was assessed using the Quality Assessment Tool for Observational Cohort and Cross-Sectional Studies (NHLBI, 2017), which assesses 14 items, rating quality as poor, fair or good.

## Results

### Search results

**Fig. 1.**
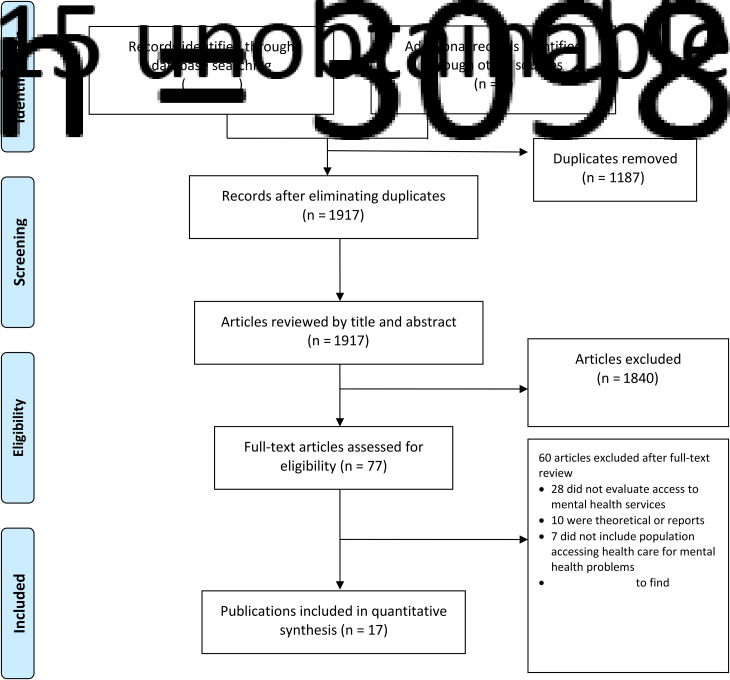
Flow chart of articles included and excluded after the systematic review.

The search strategy produced 3098 potentially relevant studies (see Fig. 1 PRISMA flow diagram). Further six articles were identified from the references of the articles selected. Of these, 1187 were duplicates. Of those remaining, 1840 were excluded after reviewing the title and abstract. After reviewing the full text of the remaining articles, 60 were excluded for the following main reasons: 28 did not evaluate access or use of mental health services, ten were reports or theoretical articles, and seven did not include population accessing health care for mental health problems. Finally, 17 articles were selected.

The data of the included studies were extracted and summarised (see Table 2). Five were repeated cross-sectional studies, as well as four time series studies, three ecological studies, three cohorts, one panel study and one longitudinal study. Eleven studies employed national population samples and six employed regional samples. The studies were based on samples from European countries (58.82%), USA (29.41%), Australia (5.88%) and Republic of China (5.88%).

### Study quality

The results of the quality assessment of the included studies are presented in Supplementary Table S2. All studies have an objective clearly stated and a study population prespecified. Only two studies provided a sample size justification (Buffel *et al*., 2015; Chen and Dagher, 2016). Nine studies measured the exposure of interest prior to the outcome (Córdoba-Doña *et al*., 2014; Wong *et al*., 2014; Bidargaddi *et al*., 2015; Gotsens *et al*., 2015; Modrek *et al*., 2015; Chen and Dagher, 2016; Dunlap *et al*., 2016; Hawton *et al*., 2016; Petrou, 2017). Seven studies did not provide adjustment for confounding variables (Ostamo and Lönnqvist, 2001; Iglesias García *et al*., 2014; Bidargaddi *et al*., 2015; Sicras-Mainar and Navarro-Artieda, 2015; Ásgeirsdóttir *et al*., 2017; Hawton *et al*., 2016; Petrou, 2017).

### Impact of the economic crisis on the use of health facilities

#### Use of general and specialised care for mental health problems

Periods of economic crisis appeared linked to an increase of general help seeking for mental health problems (Bidargaddi *et al*., 2015; Buffel *et al*., 2015; Sicras-Mainar and Navarro-Artieda, 2015), with mixed patterns for the use of specialised psychiatric care (Iglesias García *et al*., 2014; Wong *et al*., 2014; Buffel *et al*., 2015; Modrek *et al*., 2015; Chen and Dagher, 2016; Petrou, 2017). Two studies conducted in European Union countries found that a significantly increased contact with a general practitioner for mental health problems occurred during the period of economic crisis (Buffel *et al*., 2015; Sicras-Mainar and Navarro-Artieda, 2015), but a significantly decreased contact with a psychiatrist (Buffel *et al*., 2015) or no change in the use of specialist care occurred despite the higher proportion of referrals (Sicras-Mainar and Navarro-Artieda, 2015). Bidargaddi *et al*. (2015) found a higher number of visits to the medical emergency room for reasons of mental health during economic slow-down. Two studies in the USA found a significant increase in the utilisation of mental health services during the Great Recession (Wong *et al*., 2014; Modrek *et al*., 2015), whereas two others found a significant decrease in the use of mental health services in this period (Iglesias García *et al*., 2014; Chen and Dagher, 2016), and one found no impact of the crisis on the utilisation rate (Petrou, 2017).

#### Hospitalisations for mental health problems

Several studies found an increase of hospital admissions for mental disorders during periods of economic crisis (Korkeila *et al*., 1998; Modrek *et al*., 2015; Bonnie Lee *et al*., 2017). In Finland (Korkeila *et al*., 1998), the increase of admissions was particularly significant in multiple readmissions among new inpatients (from 0.05 to 0.08, *p* < 0.001), and in the diagnostic group of mood disorders among first-timers (by 60%) and among patients with previous admissions (by 39%). In the USA (Modrek *et al*., 2015), a marginally significant increase in the postrecession trend in inpatient utilisation compared with prerecession trend (*b*  =  0.002; *p*  =  0.078) was found. In Taiwan, Bonnie Lee *et al*. (2017) found increased rates of hospitalisation for depressive illnesses. Specifically, the AIR of hospital admissions among the low-income group were ten times higher than those of the high-income group.

#### Use of health facilities due to suicide behaviour

The studies included found mixed results regarding the use of mental health care due to suicide behaviour (Ostamo and Lönnqvist, 2001; Córdoba-Doña *et al*., 2014; Hawton *et al*., 2016; Ásgeirsdóttir *et al*., 2017). Studies conducted in Nordic countries (Finland and Iceland) showed no overall increase in attendance rates due to suicide attempts and self-harm following economic crises (Ostamo and Lönnqvist, 2001; Ásgeirsdóttir *et al*., 2017). On the contrary, an increase in the use of health facilities for suicide attempt and self-harm after the onset of the 2008 crisis was found in other European countries (Spain and England) (Córdoba-Doña *et al*., 2014; Hawton *et al*., 2016), with authors proposing that the increase may be related to changes in unemployment.

### Impact of the economic crisis on the use of prescription drugs

Different studies found an increase in the use of psychotropic drugs during the period of an economic crisis, including psychotropic medications to treat depressive and anxiety disorders (Gotsens *et al*., 2015; Modrek *et al*., 2015; Sicras-Mainar and Navarro-Artieda, 2015; Chen and Dagher, 2016). Gotsens *et al*. (2015) compared the use of psychotropic drugs between natives and immigrants who arrived in Spain before 2006 and found that the increase in the use of psychotropic drugs was higher among immigrant men.

### Impact of macroeconomic indicators on the use of mental health care

Several articles included in this review found that unemployment rates were associated with changes in help-seeking behaviour for mental health problems (Córdoba-Doña *et al*., 2014; Iglesias García *et al*., 2014; Wong *et al*., 2014; Bidargaddi *et al*., 2015; Buffel *et al*., 2015; Modrek *et al*., 2015; Dunlap *et al*., 2016; Ásgeirsdóttir *et al*., 2017). The likelihood of men contacting a general practitioner for mental health problems was found to be higher in countries experiencing an increase in the unemployment rate (OR = 1.031, 95% CI) (Buffel *et al*., 2015). Studies in the USA found significant associations between higher local unemployment and increased outpatient visits, use of opiates and sleep aids (Modrek *et al*., 2015), as well as increased outpatient utilisation among veterans exempt from copayments (Wong *et al*., 2014). However, a third study in the USA (Dunlap *et al*., 2016) found that individuals who resided in counties with higher unemployment rates were less likely to use mental health services compared with individuals who resided in counties with the lowest unemployment rates (ARR  =  0.58, 0.62 and 0.71). Similarly, in Spain, the increase in the unemployment rate was associated with a clear decrease in mental health demand (Iglesias García *et al*., 2014). Unemployment rates were also associated with the use of mental health care due to suicide attempts in men, accounting for almost half of the cases during the five initial years of the crisis in Spain (Córdoba-Doña *et al*., 2014). However, Ásgeirsdóttir *et al*. (2017) found that a 1% increase in unemployment rate was significantly associated with reduced attendance rates due to suicide attempts among men in Iceland (RR = 0.84; 0.76–0.93), but not among women.

Buffel *et al*. (2015) found that, in countries with a decline in the GDP growth rate, employed men were less likely to contact a psychiatrist (OR = 0.966, 95% CI) compared with those in countries with an increase in the GDP growth rate, while Iglesias García *et al*. (2014) found that GDP increase was strongly associated with an increased demand for mental health care.

Finally, Dunlap *et al*. (2016) found that individuals who resided in states with higher rates of serious mortgage delinquency were less likely to use mental health services (ARR  =  0.54, 0.52 and 0.73, respectively).

### Impact of individual indicators on the use of mental health care

Several studies included showed a higher utilisation of mental health care by women (such as access to outpatient visits, emergency department, prescription drugs and hospitalisation) compared with men during the crisis (Buffel *et al*., 2015; Chen and Dagher, 2016; Bonnie Lee *et al*., 2017). Regarding the risk of attendance of health facilities due to suicidal behaviour, Ostamo and Lönnqvist (2001) showed that there was a convergence of rates between genders, with the men's rates decreasing 15% (trend test *χ*^2^  =  10.45, *p* ⩽ 0.001) and the women's rates increasing 8% (trend test *χ*^2^  =  2.55, *p*  =  0.11). Other studies showed that socioeconomic factors were more strongly associated with suicidal behaviour care in men than in women (Córdoba-Doña *et al*., 2014; Ásgeirsdóttir *et al*., 2017).

Two studies found that adults aged 35–54 years were the risk group for the use of care due to suicidal behaviour (Córdoba-Doña *et al*., 2014; Hawton *et al*., 2016).

Bonnie Lee *et al*. (2017) suggested low income to be a risk factor for hospitalisation for depressive disorders.

Gotsens *et al*. (2015) found that, in Spain, the 2008 economic crisis may have had a worse impact on the health status of immigrants, as shown by the loss of the ‘healthy immigrant effect’ and the equalisation of the previously lower use of psychotropic drugs among immigrants compared with natives. Chen and Dagher (2016) found that ethnic minorities presented lower rates of health care use during the 2008 Great Recession. Specifically, compared with white women, African American women had significantly fewer physician visits (IRR  =  0.71, *p*  =  0.01), and Latinas and African American women used significantly fewer prescription drugs (IRR  =  0.75, *p* < 0.001; IRR  =  0.71, *p* < 0.001). Compared with white men, Latino men had significantly lower rates of physician visits (IRR  =  0.72, *p* < 0.05), and lower rates of prescription drug utilisation (IRR  =  0.72, *p* < 0.001). Burgard and Hawkins (2014) found that levels of foregone mental health care rose in the Great Recession of 2007–2009, but that disparities between ethnic groups in foregone mental care were stable during the recession.

## Discussion

To our knowledge, this is the first systematic review to specifically study the impact of the economic crisis on the utilisation of mental health care following PRISMA guidelines. This study is relevant for systematising the scarce literature available, and for highlighting the risk of a growing treatment gap, particularly among the most vulnerable groups.

Our results suggest an increase of general help-seeking behaviour for mental health problems, with more contradictory results in relation to the use of specialised psychiatric care. There may be several explanations for these findings. First, in times of economic crisis, more accessible and affordable general health care might be the preferred pathway to care, with the subsequent increase in unmet need for specialised care (Buffel *et al*., 2015). In these periods, reduced mental health budgets may decrease availability of mental health services and/or they may be unaffordable because of the reduction of households’ disposable income, lack of health insurance coverage or the introduction of copayment in the public health care sector (Dunlap *et al*., 2016). Second, decreased motivation to demand specialised care may be due to possible negative consequences, such as fear of losing a job due to work disability or treatment stigma (Iglesias García *et al*., 2014). Lastly, the adverse social circumstances that occur in periods of economic crisis might cause health expectations to decrease and induce more personal efforts to be taken on to achieve these expectations (Iglesias García *et al*., 2014).

The review conducted by Martin-Carrasco *et al*. (2016) had already described an increase in the treatment gap during times of economic crisis, pointing out the lack of accessibility to services, the austerity measures and the increased stigma as probable explanations.

Our review found different trends in relation to the use of mental health care due to suicide behaviour between the Nordic countries and other European countries. Possible factors explaining these findings might be Nordic countries’ relatively high levels of social capital and strong welfare systems, possibly mitigating the adverse consequences of unemployment on suicidal outcomes (Ostamo and Lönnqvist, 2001; Ásgeirsdóttir *et al*., 2017).

Our results also suggest that economic crises might be associated with a higher use of prescription drugs and an increase in hospital admissions for mental disorders, as had been found in previous reviews (Zivin *et al*., 2011; Cheung and Marriott, 2015).

The results provide information on the patterns of demand for care of different groups defined by an axis of inequality during economic crises. The groups of people most susceptible to the effects of crises were not consistently those that most accessed mental health care (Burgard and Hawkins, 2014; Córdoba-Doña *et al*., 2014; Iglesias García *et al*., 2014; Wong *et al*., 2014; Bidargaddi *et al*., 2015; Buffel *et al*., 2015; Gotsens *et al*., 2015; Modrek *et al*., 2015; Dunlap *et al*., 2016; Chen and Dagher, 2016; Ásgeirsdóttir *et al*., 2017; Bonnie Lee *et al*., 2017). Mental health care utilisation patterns depend on the recognition that help is needed (Andrade *et al*., 2008; Mojtabai *et al*., 2002), on structural factors including financial costs (Mojtabai, 2009), and availability of services (Wells *et al*., 2002; Saxena *et al*., 2003), and on attitudinal factors (Sareen *et al*., 2007; Mojtabai, 2010). These factors might change during economic crises and affect differently the various socioeconomic groups, possibly exacerbating systemic problems in access to care and widening social inequalities in mental health. The reasons for the increased treatment gap among vulnerable groups might include a disproportionate worsening of socioeconomic conditions, the impact of austerity measures, and subsequent reduced available income, lack of social protection and a reduction in available health care, but also worse perceived need for care, reluctance to seek services and/or cultural or linguistic barriers.

In the studies included, during periods of crisis, women used mental health care more frequently than men (Bidargaddi *et al*., 2015; Buffel *et al*., 2015; Chen and Dagher, 2016; Bonnie Lee *et al*., 2017). This might reflect women's relatively worse mental health status and higher need for care or gender differences in healthcare-seeking behaviour. Reasons for gender differences in healthcare-seeking behaviour could be greater stigma among men, a greater ability of women to identify their mental health problems or differences in health insurance coverage. Some of the studies reviewed suggest that socioeconomic factors may be more strongly associated with suicidal behaviour in men than in women (Córdoba-Doña *et al*., 2014; Hawton *et al*., 2016; Ásgeirsdóttir *et al*., 2017). One possible explanation to this finding is that men are subjected to more pressure from their working role and relative expectations of socioeconomic success, thereby sensitised to unmet expectations. Related to this, mild-adulthood seemed to be the most consistent risk group for the use of care due to suicidal behaviour (Córdoba-Doña *et al*., 2014; Hawton *et al*., 2016). This finding could be attributed to the fact that it is a period of pressure to the main earners, and during which financial crisis-related events most frequently occur.

### Policy implications

The results of this systematic review highlight the need for health services to be particularly attentive and responsive to changes in patients’ socioeconomic status, especially to the needs of the most vulnerable groups.

Models of care that are closer to the population, that facilitate the early identification of mental health problems and the implementation of integrated interventions, and that have a focus on prevention of mental health problems and disorders are particularly useful.

It is crucial to maintain universal, accessible and affordable health care of good quality to avoid increasing the treatment gap.

Additionally, reforms of social welfare to maintain or strengthen safety nets and interventions across several sectors beyond the mental health sector are fundamental to minimise increasing social inequalities in mental health during economic crises.

### Strengths and limitations

This review provides updated evidence about the impact of economic crises on the use of mental health care, and it is the first systematic review following the PRISMA statement in this field.

The results give us some information on the patterns of demand for care of different socioeconomic groups during these periods, and we propose some insights for these findings.

However, results should be taken with caution due to several aspects.

First, studies from some of the most severely affected countries by economic crises were not available, and this low representation of geographical and health systems limits the interpretation of our results. The scarce variety of a study's origin may reflect different levels of cross-country research or a publication bias, due to financial constraints, methodological difficulties or language barriers. It would be highly beneficial if this gap in the existing literature could be improved.

Second, the diversified designs of the included studies make it difficult to derive more homogeneous and robust conclusions, and to ascertain causality. Future studies should combine both aggregate-level and individual-level research, and longitudinal studies are needed. Measurement error may have occurred for some of the indicators. Service indicators are dependent on the nature and structure of the services, many are clinical and not based on standardised interviews and have limitations such as potential variations in the registry. Most of the studies reported that, during the period studied, there was no variation in the organisation of mental health services or registration process, in addition to those resulting from the austerity measures. Due to the special features of economic crises in each country, the specific national welfare and health systems and the countries’ policies adopted to deal with the crisis, external validity may be limited, and further research is needed to confirm the results obtained.

Third, the literature included results linked to different economic crises worldwide. Different types of economic crises will influence the length, depth and effects of the recession.

## Conclusions

Research is scarce on the impact of economic crises on the use of mental health care, and methodologies used in these papers were prone to substantial bias. However, the evidence suggests that periods of economic crisis might be linked to an increase of demand for care at the general care level, an increase of hospital admissions for mental disorders and a significant higher use of prescription drugs, with more conflicting results in the use of specialised psychiatric care.

Other crises will occur in the future, and more empirical and long-term studies are needed in order to adapt mental health care systems to the needs of the populations, especially in times of economic crisis.

## Availability of data and materials

The data regarding the process of screening and selection of the articles included in this systematic review are available in an online Supplementary material.

## Supplementary material

For supplementary material accompanying this paper visit http://dx.doi.org/10.1017/S2045796018000641. click here to view supplementary material
