# Supplementary material for: Impact of economic crises on mental health care: a systematic review
Source: Epidemiol Psychiatr Sci. 2018 Nov 13;29:e7. doi: 10.1017/S2045796018000641 (PMC8061146; doi:10.1017/S2045796018000641)
Supplement: Supplementary file 1 [file epssup.zip › S2045796018000641sup001.docx]

Table S1. Search strategy for Ovid MedLine

| Identifies economic crisis TI,AB | |
| --- | --- |
| 1 | “economic recession”.ti,ab. |
| 2 | “financial crisis”.ti,ab. |
| 3 | “financial crises”.ti,ab. |
| 4 | “economic crisis”.ti,ab. |
| 5 | austerity.ti,ab. |
| 6 | unemployment.ti,ab. |
| 7 | poverty |
| 8 | 1 or 2 or 3 or 4 or 5 or 6 or 7 |
| Identifies use of mental health services TI,AB | |
| 9 | “mental health service”.ab,ti. |
| 10 | “use of services”.ab,ti. |
| 11 | “services use”.ab,ti. |
| 12 | “utilization of services”.ti,ab. |
| 13 | “services utilization”.ti,ab. |
| 14 | “primary care”.ti,ab. |
| 15 | (“health care” OR healthcare).ti,ab. |
| 16 | (“health utilization” OR “health utilisation”).ab,ti. |
| 17 | “psychiatric care”.ab,ti. |
| 18 | (“specialized care” OR “specialised care”).ab,ti. |
| 19 | “health services accessibility”.ti,ab. |
| 20 | “mental health care”.ti,ab. |
| 21 | “emergency service*”.ab,ti. |
| 22 | “outpatient service*”.ab,ti. |
| 23 | “outpatient visit*”.ab,ti. |
| 24 | “psychiatric admission*”.ab,ti. |
| 25 | “psychotropic mediation”.ab,ti. |
| 26 | 9 or 10 or 11 or 12 or 13 or 14 or 15 or 16 or 17 or 18 or 19 or 20 or 21 or 22 or 23 or 24 or 25 |
| Identifies mental health problems TI,AB | |
| 27 | “mental disorder*”.ab,ti. |
| 28 | “mental illness”.ab,ti. |
| 29 | depress*.ab,ti. |
| 30 | anxiety.ab,ti. |
| 31 | “substance abuse”.ab,ti. |
| 32 | “alcohol abuse”.ab,ti. |
| 33 | psychosis.ab,ti. |
| 34 | suicid*.ti,ab. |
| 35 | self-harm.ab,ti. |
| 36 | parasuicide.ab,ti. |
| 37 | 27 or 28 or 29 or 30 or 31 or 32 or 33 or 34 or 35 or 36 |
|  | 8 and 26 and 37 |
