# Supplementary material for: Impact of economic crises on mental health care: a systematic review
Source: Epidemiol Psychiatr Sci. 2018 Nov 13;29:e7. doi: 10.1017/S2045796018000641 (PMC8061146; doi:10.1017/S2045796018000641)
Supplement: Supplementary file 1 [file epssup.zip › S2045796018000641sup002.docx]

Table S2. Quality assessment

| **Study** | **Objective clearly stated** | **Study population specified and defined** | **Participation rate at least 50%** | **Recruitment from the same or similar populations** | **Sample size justification, power description, or variance and effect estimated** | **Exposure of interest measured prior to the outcome** | **Sufficient timeframe** | **Examination of different levels of the exposure** | **Exposure measures clearly defined, valid, reliable, and implemented** | **Exposure assessed more than once** | **Outcome assessors blinded** | **Follow up incomplete data <20%** | **Adjustment for cofounding variables** |
| --- | --- | --- | --- | --- | --- | --- | --- | --- | --- | --- | --- | --- | --- |
| Ásgerisdóttir et al., 2016 | Yes | Yes | Yes | Yes | No | No | Yes | No | Yes | Yes | N/A | N/A | No |
| Bidargaddi et al., 2015 | Yes | Yes | Yes | Yes | No | Yes | Yes | Yes | Yes | Yes | N/A | N/A | No |
| Bonnie Lee et al., 2017 | Yes | Yes | Yes | Yes | No | No | Yes | Yes | Yes | Yes | N/A | N/A | Yes |
| Buffel et al., 2015 | Yes | Yes | Yes | Yes | Yes | No | Yes | Yes | Yes | Yes | N/A | N/A | Yes |
| Burgard et al., 2014 | Yes | Yes | Yes | Yes | No | No | Yes | Yes | Yes | Yes | N/A | N/A | Yes |
| Chen & Dagher, 2014 | Yes | Yes | Yes | Yes | Yes | Yes | Yes | Yes | Yes | Yes | N/A | No | Yes |
| Córdoba-Doña et al., 2014 | Yes | Yes | Yes | Yes | No | Yes | Yes | Yes | Yes | Yes | N/A | N/A | Yes |
| Dunlap et al., 2016 | Yes | Yes | Yes | Yes | No | Yes | Yes | Yes | Yes | Yes | N/A | N/A | Yes |
| Gotsens et al., 2015 | Yes | Yes | Yes | Yes | No | Yes | Yes | Yes | Yes | Yes | N/A | N/A | Yes |
| Hawton et al., 2016 | Yes | Yes | Yes | Yes | No | Yes | Yes | Yes | Yes | Yes | N/A | N/A | No |
| Iglesias et al., 2014 | Yes | Yes | N/A | Yes | No | No | Yes | Yes | Yes | Yes | N/A | N/A | No |
| Korkeila et al., 1998 | Yes | Yes | Yes | Yes | No | No | Yes | No | Yes | Yes | N/A | N/A | Yes |
| Modrek et al., 2015 | Yes | Yes | Yes | Yes | No | Yes | Yes | Yes | Yes | Yes | N/A | N/A | Yes |
| Ostamo & Lonnqvist, 2001 | Yes | Yes | N/A | Yes | No | No | Yes | No | Yes | Yes | N/A | N/A | No |
| Petrou, 2017 | Yes | Yes | N/A | Yes | No | Yes | Yes | No | Yes | No | N/A | N/A | No |
| Sicras-Mainar & Navarro-Arteida, 2016 | Yes | Yes | Yes | Yes | No | No | Yes | No | Yes | No | N/A | N/A | No |
| Wong et al., 2014 | Yes | Yes | Yes | Yes | No | Yes | Yes | Yes | Yes | Yes | N/A | N/A | Yes |
